# Predicting Hypoxia Using Machine Learning: Systematic Review

**DOI:** 10.2196/50642

**Published:** 2024-02-02

**Authors:** Lena Pigat, Benjamin P Geisler, Seyedmostafa Sheikhalishahi, Julia Sander, Mathias Kaspar, Maximilian Schmutz, Sven Olaf Rohr, Carl Mathis Wild, Sebastian Goss, Sarra Zaghdoudi, Ludwig Christian Hinske

**Affiliations:** 1 Digital Medicine, University Hospital of Augsburg, Augsburg, Germany; 2 Hematology and Oncology, University Hospital of Augsburg, Augsburg, Germany; 3 Gynecology and Obstetrics, University Hospital of Augsburg, Augsburg, Germany; 4 Department of Anaesthesiology, LMU University Hospital, LMU Munich, Munich, Germany

**Keywords:** artificial intelligence, machine learning, hypoxia, hypoxemia, anoxia, hypoxic, deterioration, oxygen, prediction, systematic review, review methods, review methodology, systematic, hospital, predict, prediction, predictive

## Abstract

**Background:**

Hypoxia is an important risk factor and indicator for the declining health of inpatients. Predicting future hypoxic events using machine learning is a prospective area of study to facilitate time-critical interventions to counter patient health deterioration.

**Objective:**

This systematic review aims to summarize and compare previous efforts to predict hypoxic events in the hospital setting using machine learning with respect to their methodology, predictive performance, and assessed population.

**Methods:**

A systematic literature search was performed using Web of Science, Ovid with Embase and MEDLINE, and Google Scholar. Studies that investigated hypoxia or hypoxemia of hospitalized patients using machine learning models were considered. Risk of bias was assessed using the Prediction Model Risk of Bias Assessment Tool.

**Results:**

After screening, a total of 12 papers were eligible for analysis, from which 32 models were extracted. The included studies showed a variety of population, methodology, and outcome definition. Comparability was further limited due to unclear or high risk of bias for most studies (10/12, 83%). The overall predictive performance ranged from moderate to high. Based on classification metrics, deep learning models performed similar to or outperformed conventional machine learning models within the same studies. Models using only prior peripheral oxygen saturation as a clinical variable showed better performance than models based on multiple variables, with most of these studies (2/3, 67%) using a long short-term memory algorithm.

**Conclusions:**

Machine learning models provide the potential to accurately predict the occurrence of hypoxic events based on retrospective data. The heterogeneity of the studies and limited generalizability of their results highlight the need for further validation studies to assess their predictive performance.

## Introduction

A key factor in risk assessment for sequelae and mortality in hospitalized patients is hypoxia. It describes the decreased availability of oxygen in specific body regions (tissue hypoxia) or in the body as a whole (general hypoxia) [1-3]. To prevent general hypoxia and to detect deterioration quickly, hypoxemia monitoring is commonly performed using pulse oximetry as a continuous and noninvasive assessment, especially in the intensive care unit (ICU) and operating room (OR) [4]. Hypoxemia is defined as an abnormally low level of blood oxygen. In addition to pulse oximetry, it can be assessed through an arterial blood gas analysis or imaging techniques, which can additionally serve as reliable indicators of subsequent tissue damage [3]. A multinational, multicenter study including 117 ICUs found a hypoxemia prevalence of more than 50% among all ICU patients [5]. The severity of hypoxemia was shown to be a direct risk factor for mortality in patients with hypoxemia. Being able to validly assess the individual risk of future hypoxemic and ultimately hypoxic events is therefore highly relevant.

To determine the risk or stage of a disease, artificial intelligence (AI) has been increasingly introduced into clinical routine in recent years to exploit underlying causal mechanisms that may not be accessible to humans. As a prime example, machine learning (ML) as a discipline of AI is being successfully used for cancer tissue classification in medical imaging [6,7]. ML is also already being applied for prognostic purposes, for example, in the examination of patient characteristics to identify an increased risk of deterioration tendencies such as atrial fibrillation and of developing sequelae of diabetes mellitus or hereditary diseases [8-10].

Efforts to date of using ML to predict hypoxic events are being conducted in a variety of settings and demonstrate diverse approaches and methodologies. Studies differ significantly in terms of the patient population assessed, definition of prediction outcome, features used to predict hypoxia, and ML algorithms used, thus increasing the difficulty to generalize the conclusions of individual studies. It is therefore challenging to compare and evaluate these studies comprehensively.

This review aimed to provide a systematic and structured overview of the existing approaches to predict hypoxic events in the hospital setting. Our specific objectives were to summarize the different populations, model details, and prediction performance to capture the current state of available models; identify gaps and limitations; highlight promising approaches and methodologies; and provide guidance for future research in this area.

## Methods

### Protocol

This review was reported in accordance with the PRISMA (Preferred Reporting Items for Systematic Reviews and Meta-Analyses) statement (Checklist 1) [11]. The protocol was registered in the International Prospective Register of Systematic Reviews (PROSPERO) prior to data extraction (reference CRD42023381710).

### Search Strategy

Relevant literature was searched for using Ovid with Embase and MEDLINE, Web of Science, and Google Scholar. Although the prior 2 databases were searched via their web query interface, Google Scholar was searched using the software Publish or Perish, as it allows for more complex queries [12].

Publications on the topic of hypoxia prediction using ML were searched by creating 2 sets of search terms, with the first set addressing hypoxia (including hypoxemia) and the second set addressing ML. With the identified search engines, the intersection of these 2 groups was then searched for, adjusting the syntax according to the search logic of the respective search engine. If Medical Subject Headings or thesaurus entries were available, the selected terms were included in the search logic accordingly. For the searches using Ovid and Web of Science, the search results were filtered to only include studies that did not use wearables for data collection and that were published in the English and German languages. Those filters were not applicable for the search of Google Scholar using Publish or Perish.

The selection and deduplication process was performed using Covidence (Veritas Health Innovation Ltd), with undetected duplicates removed by hand [13]. The search results of all databases were included, and duplicates were removed. The abstracts of the remaining results were independently screened by 2 reviewers. Results that met the selection criteria were reviewed in their entirety for the assessment of eligibility by 2 reviewers. In addition, references of the included studies were also screened for studies that meet the inclusion criteria and were subsequently included where appropriate. The search strategy was developed by 1 team member and reviewed by another with expertise in conducting systematic reviews. The detailed search strategy can be found in Multimedia Appendix 1.

### Selection Criteria

Primary outcomes were model features, definition of the prediction end point, and predictive performance. Studies developing ML models to predict hypoxia or hypoxemia in continuously monitored human inpatients were included. Both studies of patients who were mechanically ventilated and spontaneously breathing were included. Hypoxia could be a main outcome or an auxiliary goal.

Studies that assessed hypoxia only in specific tissues were excluded, as this review addresses the prediction of general hypoxia as an important indicator of critical illness for risk stratification and early detection of patients at risk of acute health deterioration. Additionally, studies focusing on a population <18 years of age were not included, since the distinct etiologies, risk factors, and clinical presentations of hypoxia in pediatric patients may limit the generalizability of the findings to the population of adult inpatients.

The definition of the end point of hypoxia prediction (eg, specific oximetry thresholds or time frames of prediction) was left unspecified due to the expected heterogeneity in the approaches. The patient population of the included studies was not limited to a specific hospital setting or ward.

### Data Extraction and Risk of Bias

Data extracted included the data source; sample size and setting; model variables; prediction end point and time frame; type of model; and the predictive performance of each model, usually expressed as classification measures such as sensitivity, specificity, positive predictive value (PPV), negative predictive value (NPV), or area under the receiver operating characteristics (AUROC). Missing values of performance measures and summary data influenced the risk-of-bias assessment.

A qualitative synthesis of the included studies was conducted. For this purpose, an overview of all studies was provided in a narrative summary by categorizing them into subgroups based on the population, model features, model types, and setting. For each study, the model with the highest performance according to performance metrics was selected to summarize AUROC, sensitivity, specificity, PPV, and NPV as the most reported performance measures. In the case of studies that examined multiple prediction outcomes, the outcome definition that is the most similar to those of the other studies was chosen for reporting. For studies reporting 1 performance value per patient, a mean value was calculated for each measure. Because of the heterogeneous study designs and characteristics of the data used, as well as missing summary data of model performances, conducting a meta-analysis was not feasible.

To assess the risk of bias, quality, and applicability of the studies included, Prediction Model Risk of Bias Assessment Tool (PROBAST) was used [14]. This tool is specifically designed to investigate the quality of prediction models and has become increasingly prevalent in systematic reviews in recent years. Assessment outcomes were evaluated based on 4 segments—participants, predictors, outcome, and analysis—and were determined by a comprehensive questionnaire. Risk of bias was rated as high, low, or unclear. If 1 domain suggested a high risk of bias, the overall risk of bias for that study was considered high. The assessment was conducted by a single researcher, with a second researcher reviewing the process independently.

## Results

### Literature Search

**Figure 1.**
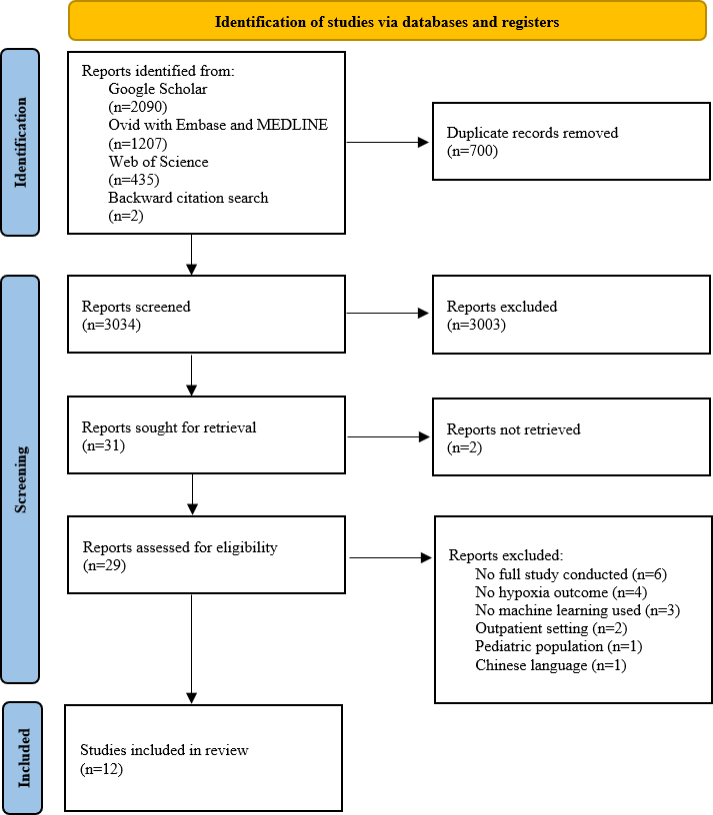
PRISMA (Preferred Reporting Items for Systematic Reviews and Meta-Analyses) flow diagram.

The initial search retrieved a total of 3734 studies (Figure 1). After removing a total of 700 duplicates, title and abstract screening identified the full texts of 31 studies for the assessment of eligibility. Of these, 19 studies were excluded due to not being a full study (n=6), not assessing a hypoxia outcome (n=4), not using machine learning (n=3), inability to obtain the full text (n=2), having an outpatient setting (n=2), having a pediatric patient population (n=1), and being in the Chinese language (n=1). The remaining 12 studies were included in the review.

### Study Characteristics

#### Overview

Table 1 presents the characteristics of all included studies and gives an overview of the best-performing model in each study, divided into conventional ML and deep learning models for studies including both. The studies were conducted in the United States [15-22], China [23,24], Germany [25], and the United Arab Emirates [26]. Half (6/12, 50%) of them were published after 2020 [15,16,19,21,22,26]. In 3 (25%) of the 12 studies, the prediction of hypoxia was a side or auxiliary goal [17,19,21], whereas it was the main study aim for the other studies.

**Table 1.** Study characteristics of the reviewed studies (n=12). The model with the highest performance in each study is reported. For studies using both conventional machine learning and deep learning models, each best-performing model is reported. For studies examining multiple prediction outcomes, the outcome definition that is the most similar to those of other studies was chosen for reporting. For studies reporting 1 performance value per patient, a mean value was calculated.

Reference	Sample size n	Clinical variables, n	Prediction end point	Model	Performance	External validation
Annapragada et al [15] (2021)	2435	1	SpO_2_^a^ <92% within the next 5 and 30 min (occurrence and magnitude of hypoxemic events)	LSTM^b^	PPV^c^: 0.94 Sensitivity: 0.80 Specificity: 0.99	Yes
Chen et al [16] (2021)	57,171	21	SaO_2_^d^ <93% within the next 5 min	GBT^e^	AUROC^f^: 0.89	Yes
ElMoaqet et al [17] (2014)	119	1	SpO_2_ ≤89% within the next 20 and 60 s	Lin^g^	AUROC: 0.93	No
Erion et al [18] (2017)	57,173	1	SpO_2_ ≤92% within the next 5 min	LSTM GBT	LSTM AUROC: 0.87 GBT AUROC: 0.86	No
Geng et al [23] (2018)	308	3	SpO_2_ <90% for any duration during the endoscopic procedure	LR^h^	AUROC: 0.76	No
Geng et al [24] (2019)	220	3	SpO_2_ <90% for any duration during the endoscopy procedure	ANN^i^	AUROC: 0.80	No
Lam et al [19] (2022)	39,630	26	SpO_2_ <91% and <96% after algorithm evaluation and any time during hospitalization	XGB^j^ RNN^k^	XGB AUROC: 0.64 RNN AUROC: 0.64	Yes
Lundberg et al [20] (2018)	36,232	>65	SpO_2_ ≤92% initial status and within the next 5 min	GBM^l^	AUROC: 0.90	No
Ren et al [21] (2022)	17,818	3	PaO_2_^m^/FiO_2_^n^ ≤150 at any time during ventilation	NN^o^ LR	NN AUROC: 0.83 LR AUROC: 0.81	Yes
Sippl et al [25] (2017)	620	17, RF^p^ and NN used subsets of 6 and 7	Presence and severity of temporary oxygen desaturation during anesthesia induction and intubation based on expert annotations	NN RF	NN sensitivity: 0.74 NN specificity: 0.93 RF sensitivity: 0.35 RF specificity: 0.99	No
Statsenko et al [26] (2022)	605	2D and 3D diagnostic images of the chest	Markers of systemic oxygenation: functional (HR^q^, BR^r^, SBP^s^, and DBP^t^) and biochemical findings (SpO_2_, serum potassium level, and AG^u^)	CNN^v^	MAE^w^: mean 7.941% (SD 4.131%)	No
Xia et al [22] (2022)	14,777	29	PaO_2_ <60 mm Hg after extubating	RF	AUROC: 0.792	No

a SpO_2_: peripheral oxygen saturation.

b LSTM: long short-term memory.

c PPV: positive predictive value.

d SaO_2_: arterial oxygen saturation.

e GBT: gradient boosted tree.

f AUROC: area under the receiver operating characteristics.

g Lin: linear regression.

h LR: logistic regression.

i ANN: artificial neural network.

j XGB: extreme gradient boosting.

k RNN: recurrent neural network.

l GBM: gradient boosting machine.

m PaO_2_: partial pressure of oxygen.

n FiO_2_: fraction of inspired oxygen.

o NN: neural network.

p RF: random forest.

q HR: heart rate.

r BR: breath rate.

s SBP: systolic blood pressure.

t DBP: diastolic blood pressure.

u AG: anion gap.

v CNN: convolutional neural network.

w MAE: mean averaged error to the range of values.

#### Data Sources and Population

Most studies (9/12, 75%) analyzed a large sample size of 500 or more patients [15,16,18-22,25,26]. Data from the publicly available databases Medical Information Mart for Intensive Care and eICU Collaborative Research Database were used in 4 of the studies [15,16,21,22], whereas 3 studies relied on data collected via an anesthesia information management system (AIMS) [16,18,20]. AIMSs are widely adopted hardware and software solutions that are integrated into a hospital’s electronic health record system and are used to manage and document a patient’s perioperative measurements [27,28]. The studies were set in the OR (n=5) [16,18,20,23,24], the ICU (n=3) [15,21,22], and mixed or general care units (n=4) [17,19,25,26]. Of the 12 studies analyzed, 10 (83%) did not include patients with COVID-19 [16-25], whereas the remaining 2 (17%) studies either were performed only on patients who tested positive for COVID-19 or were externally validated on a COVID-19 cohort [15,26].

#### ML Model Specifics

**Figure 2.**
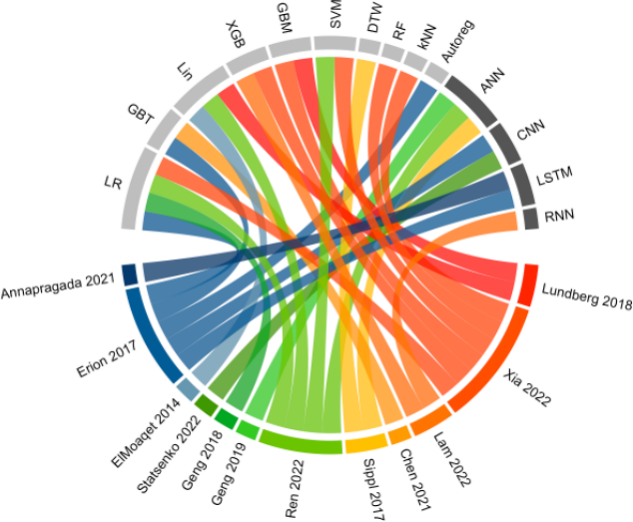
Machine learning (ML) methods used by each study. ML methods (upper half) in gray: conventional ML; ML methods in black: deep learning. Studies are sorted by the number of clinical variables used. Studies in blue: 1 clinical variable; studies in green: 2-5 clinical variables; studies in yellow to red: >5 clinical variables. ANN: artificial neural network; Autoreg: autoregressive model; CNN: convolutional neural network; DTW: dynamic time warping; GBM: gradient boosting machine; GBT: gradient boosted tree; kNN: k-nearest neighbor; Lin: linear regression; LR: logistic regression; LSTM: long short-term memory; RF: random forest; RNN: recurrent neural network; SVM: support vector machine; XGB: extreme gradient boosting.

Figure 2 [15-26] gives an overview of the models and the number of variables used in each study. Exclusively conventional ML algorithms were applied in 5 of the identified studies [16,17,20,22,23], whereas 7 studies included deep learning algorithms [15,18,19,21,24-26]. Models based on logistic regression were used most often (n=4) [18,21-23], followed by artificial neural networks (n=3) [21,24,25].

The number of clinical variables included ranged from 1 to over 65 different variables. The prediction of hypoxic events was based solely on prior peripheral oxygen saturation (SpO_2_) values in 3 studies [15,17,18], whereas 4 studies used 2 or 3 clinical variables as input [21,23,24,26]. The remaining 5 studies relied on at least 6 variables [16,19,20,22,25]. The most frequently used variable sources were oximetry measurements (9/12, 75%) [15-22,25] and static patient characteristics such as age (5/12, 42%) [16,19,20,23,25]. Additionally, a single study relied on diagnostic images of the chest to make predictions [26].

The prediction end point was defined by a threshold of SpO_2_ between 89% and 92% for most of the studies (7/12, 58%) [15,17-20,23,24]. Thresholds of the partial pressure of oxygen, the arterial oxygen saturation, or the ratio of partial pressure of oxygen to the fraction of inspired oxygen were used in 3 other studies [16,21,22]. The remaining 2 studies assessed the presence and severity of hypoxia as defined by expert annotations and predicted functional markers of hypoxia, respectively [25,26]. Defined time frames for prediction included the length of a certain procedure [21,23-25], any time after extubating [22], and a set time window of 5 to 30 minutes [15-18,20].

#### Performance

**Figure 3.**
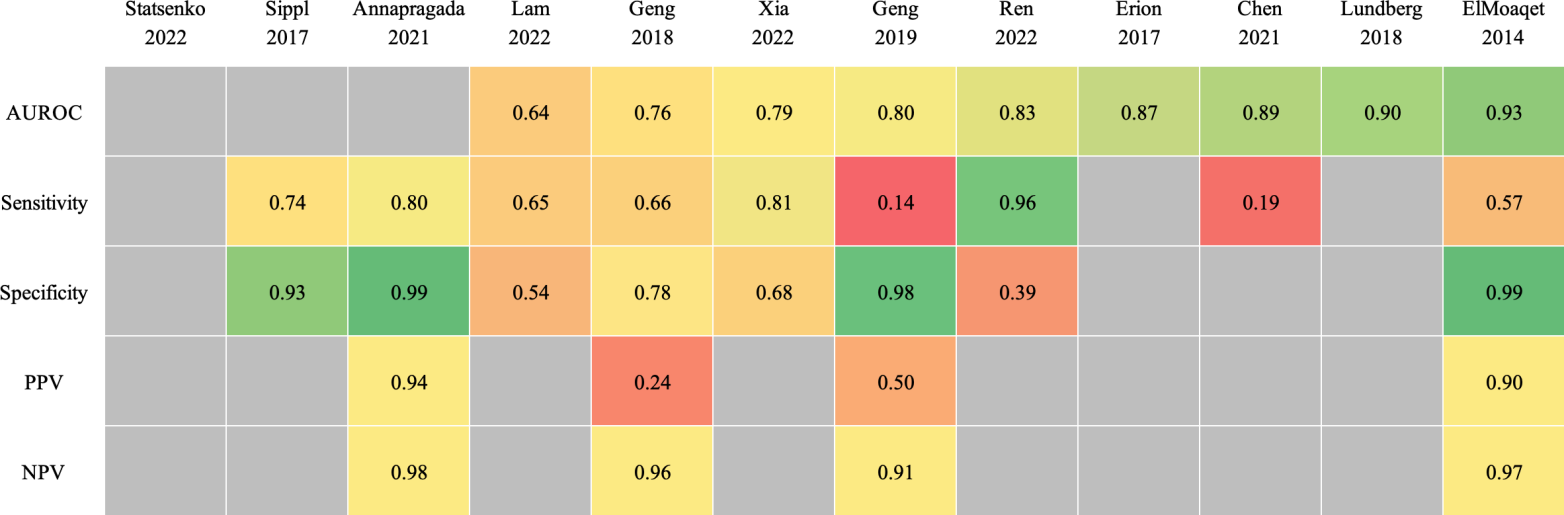
Heat map of performance measures, sorted by AUROC. The performance of the best-performing model in each study is presented. In the case of studies that examined multiple prediction outcomes, the outcome definition that is the most similar to the other studies was chosen for reporting. For studies stating 1 performance value per patient, the metrics represent the mean value. For 3 of the included studies, hypoxia prediction was not the main study aim [17,19,21]. The reported performance measures of 1 study were based on 10 individual patients and therefore have limited informative value. One study only reported the proportion of the mean averaged error to the range of values. AUROC: area under the receiver operating characteristics; NPV: negative predictive value; PPV: positive predictive value.

Most of the 12 studies reported sensitivity (n=9, 75%), specificity (n=8, 67%), or AUROC (n=9, 75%) as classification measures. Other performance indicators were PPV, NPV, area under the precision-recall curve, accuracy, and *F*_1_-score. The most frequently reported performance measures of the best-performing model in each study are summarized in a heat map (Figure 3 [15-26]). The reported performance measures of 1 study were based on 10 individual patients since the focus of the study was to propose a performance metric and therefore have limited informative value [17]. One other study only reported the proportion of the mean averaged error to the range of values [26].

Of the 9 studies reporting AUROC, 8 (89%) showed a value higher than 0.75 [16-18,20-24]. This included 3 studies that showed a significant trade-off between sensitivity and specificity [17,21,24]. The overall performance was moderate or high with respect to classification metrics, both in studies performing the prediction task as the main study aim and in studies predicting hypoxia as a side or auxiliary goal. In studies drawing a comparison to anesthesiologist decisions, the prediction models alone or anesthesiologists using those models outperformed anesthesiologists without access to the model [18,20].

Deep learning and conventional ML are not directly comparable as they are not being applied on the same data set and the performance metrics are not consistently reported. However, in all studies comparing the 2 approaches, deep learning models showed similar or better performance than conventional ML models considering classification metrics [18,19,21,25]. Additionally, models only using prior SpO_2_ data as a variable tended to outperform models using more clinical variables [15,17,18]. Two (67%) of the 3 studies only using prior SpO_2_ data applied a long short-term memory (LSTM) algorithm, 1 of which was able to predict the detailed trend of the SpO_2_ waveform [15,18]. Multitask learning for the prediction of related end points was implemented in 1 study, showing improved performance with an increasing number of tasks [19]. Approaches for providing explainability of their prediction outcome were presented in 2 studies, with 1 offering a real-time prediction tool displaying the contributing factors of an individual patient’s hypoxemia risk within the next 5 minutes [16,20].

### Risk-of-Bias Assessment

**Figure 4.**
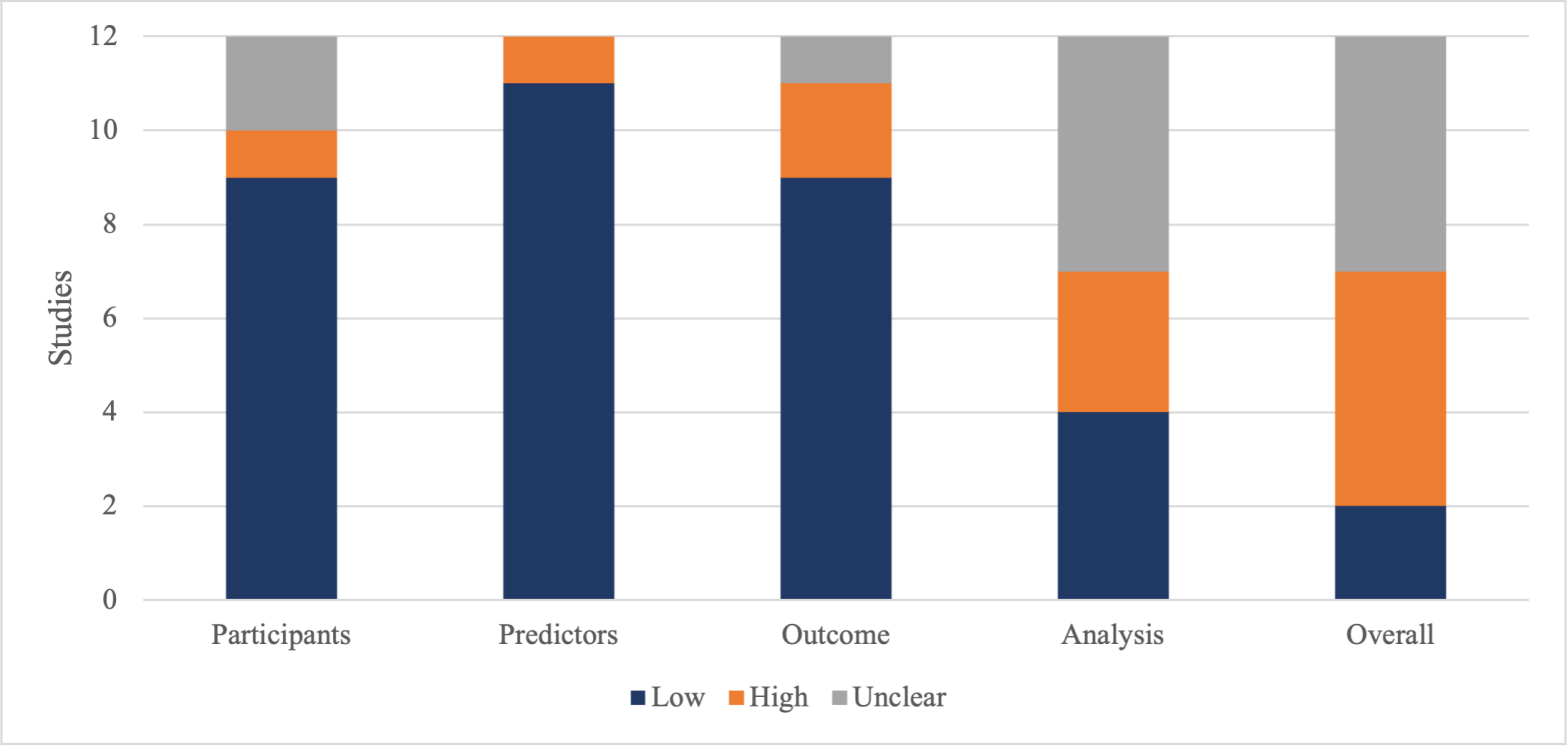
Risk-of-bias assessment for all studies (n=12) based on 4 segments. The graph shows the number of studies with low, high, and unclear risk of bias by the author’s assessment using PROBAST (Prediction Model Risk of Bias Assessment Tool).

PROBAST was used to assess the risk of bias and applicability of each study. In the case of external validation, the assessment for that validation was performed separately. An overview of the overall and segment ratings of all 12 studies analyzed are shown in Figure 4. The overall risk of bias was rated as high or unclear for most of the studies (10/12, 83%) [16-21,23-26]. Unclear or high risk of bias ratings were mainly due to missing details of the procedure as well as unclear or unfitting timing of predictors or outcomes. External validation was only performed in 4 of the studies [15,16,19,21], whereas the other 8 studies relied on internal validation, primarily using random split samples and cross-validation [17,18,20,22-26].

## Discussion

### Principal Findings

In this systematic review, we identified and summarized 12 studies predicting hypoxic events or markers for hypoxia. The approaches proved to be highly diverse both in their assessment and definition of a hypoxic outcome as well as in the variables and model types used. Therefore, the comparability between studies was limited by the high variability of approaches, such as the variety of settings involving different influences on blood oxygen saturation (eg, sedation during surgery).

The data used to develop the models were primarily obtained from publicly available databases or directly from hospitals’ AIMSs or electronic health record systems. Settings for the prediction included the OR, ICU, and general care units. The implemented ML models were based on both conventional ML and deep learning methods and assessed prediction end points defined as a threshold for blood oxygen measurements for most studies. Clinical variables used included patient characteristics, vital signs, and laboratory data. Blood oxygen data were the most applied model variables for hypoxia prediction.

The overall predictive performance of the presented models was moderate or high across the various settings. Deep learning approaches showed similar or better performance than conventional ML approaches within the same studies. Models predicting hypoxia solely based on prior oximetry data tended to outperform models using more variables as inputs, with most of these studies using an LSTM algorithm.

The demonstrated trade-off between sensitivity and specificity of model performance highlights that it may be difficult to achieve both at the same time, especially when predicting medical events. This is a major caveat that holds true for a broad variety of diagnostic tests in medicine, such as D-dimers in investigating venous thromboembolism [29]. High specificity but low sensitivity, as demonstrated by 2 of the models, might, for example, result from missing relevant variables or an insufficient number of outcome events due to small sample sizes. An algorithm with high specificity may help to reduce unnecessary interventions, potentially leading to cost savings and minimizing patient inconvenience. However, in practice, an algorithm with that trade-off does not reliably detect patients with hypoxia who require immediate attention and may therefore be more appropriate as a decision support tool rather than a stand-alone diagnostic tool.

High sensitivity but low specificity on the other hand can, for example, be caused by the inclusion of variables that are highly associated with the presence of hypoxia but are not specific to hypoxia alone, or by the model being too sensitive and thus detecting subtle changes in nonhypoxic cases that are incorrectly classified as hypoxic. Practically, such a model could result in overalerting, disqualifying it for clinical application.

The informational value of many of the studies presented was limited due to a lack of external validation. In addition, more precise classification performance metrics were often not provided, thus not allowing for a meta-analysis. Unclear ratings were mostly due to missing information, particularly in the analysis segment. Comparability between studies was limited by the high variability of approaches, such as the variety of settings involving different influences on blood oxygen saturation (eg, sedation during surgery).

### Applicability and Future Opportunities

The successful prediction of hypoxic events within a time frame of 5 or even 30 minutes into the future demonstrates the ability to provide sufficient lead time for crucial treatment interventions. Hence, these results suggest the potential of developing a helpful prediction tool, applicable in clinical practice, which complements the assessment of nurses and clinicians. Such a tool could be extended by a presentation and visualization of individual factors influencing the predicted outcome of hypoxia, as demonstrated by Lundberg et al [20]. The approach to make the model more understandable is useful both for more nuanced therapy strategies and for the general usability and acceptance of an ML tool for the prediction of hypoxia in the clinical setting.

While models with many features might have higher accuracy and might be able to capture more detailed and complex relationships between the features and the outcome of hypoxia, they also come with a higher complexity for use and are prone to overfitting [30]. Given the intended use of a predictive algorithm for making timely decisions that have immediate impact on the health status of patients, complex models with excessive features could impede their implementation in clinical practice. Additionally, utility might be reduced by patients missing 1 or more of these features. Therefore, the prediction results of LSTM models based only on previous SpO_2_ values provide a foundation for further development and refinement of models using only a few, readily available, and noninvasive respiratory variables.

The results of Lam et al [19] suggest that multitask learning may contribute to higher predictive performance on related respiratory outcomes. Therefore, an approach for parallel prediction of several relevant intensive care parameters could provide a basis for further exploration. Opportunities for combined prediction include predictive models for the necessity of changes in ventilation, in airway pressure, or for increased risk of ventilation failure [31-33]. The prediction of hypoxia could also be embedded in a more general early warning score for related outcomes, for which ML mechanisms are already being applied [19,34-36]. In addition, the development of ML prediction models in a clinical context should include consideration of recent advances for the prediction of other unrelated health parameters and outcomes to avoid a complex system of different prediction systems, thus limiting the applicability and acceptability of these efforts. Forthcoming studies in this area should strive to accurately report performance details of their models, as well as to consistently define the end point of the prediction, to allow comparison with other approaches.

### Limitations

This review focused on studies predicting hypoxic or hypoxemic events and therefore did not include studies predicting related outcomes (eg, blood oxygen saturation) without stating that aim of prediction. The comparability of predictive performance among the included studies was limited due to substantial differences in methodology, variables, and end point definition, precluding a meta-analysis from being conducted. An additional challenge arose from the fact that some studies, while including hypoxia predictions, did so as an auxiliary objective and not as their primary focus. Therefore, we focused on a qualitative summary and on demonstrating the variety of approaches taken. The generalizability of the results presented might be further restricted by the countries of origin being limited to the United States, Europe, and Asia.

### Conclusion

Despite the large methodological variance of the studies presented, this review shows promising approaches for the prediction of hypoxia status, a factor that is highly informative for changes to a patient’s state of health. Future studies must aim to improve the external validation of the predictive performance and, thus, verify the generalizability of the results to additional data sets. The applicability of validated predictive models for hypoxia risk should be proven by prospective studies in clinical practice.

## Supplementary material

10.2196/50642 Multimedia Appendix 1 Search strategy, data sources, and clinical variables.

10.2196/50642 Checklist 1 PRISMA (Preferred Reporting Items for Systematic Reviews and Meta-Analyses) checklist.

## Abbreviations

AI: artificial intelligence
AIMS: anesthesia information management system
AUROC: area under the receiver operating characteristics
ICU: intensive care unit
LSTM: long short-term memory
ML: machine learning
NPV: negative predictive value
OR: operating room
PaO_2_: partial pressure of oxygen
PPV: positive predictive value
PRISMA: Preferred Reporting Items for Systematic Reviews and Meta-Analyses
PROBAST: Prediction Model Risk of Bias Assessment Tool
PROSPERO: International Prospective Register of Systematic Reviews
SpO_2_: peripheral oxygen saturation
