# Supplementary material for: Predicting Hypoxia Using Machine Learning: Systematic Review
Source: JMIR Med Inform. 2024 Feb 2;12:e50642. doi: 10.2196/50642 (PMC10879670; doi:10.2196/50642)
Supplement: Multimedia Appendix 1 [file medinform-v12-e50642-s001.docx]

# Appendix

Supplementary Table 1: Search strategy for Ovid.

| **#** | **Searches** | **Results** |
| --- | --- | --- |
| #1 | (Hypoxia or Hypoxemia or Hypoxic or Hypoxemic or Hypoxaemia or Hypoxaemic).ti,ab. or hypoxia/ or hypoxemia/ | 369,633 |
| #2 | (Artificial Intelligence or deep learn* or artificial neural network or machine learning or shallow learn* or nearest neighbor* or decision tree* or gradient-boosted tree* or random forest* or support vector machine or reinforcement learn* or cluster analys* or hierarchical cluster* or unsupervised learn* or supervised learn*).ti,ab. or artificial intelligence/ or deep learning/ or artificial neural network/ or machine learning/ or k nearest neighbor/ or "decision tree"/ or random forest/ or support vector machine/ or "reinforcement learning (machine learning)"/ or cluster analysis/ or hierarchical clustering/ or unsupervised machine learning/ or supervised machine learning/ | 578,994 |
| #3 | #1 and #2 | 1,716 |
| #4 | #3 not wearable*.ti,ab. | 1,709 |
| #5 | limit #4 to humans | 1,240 |
| #6 | limit #5 to (english or german) | 1207 |

Search date 09/08/2022. Time range: 2000 - current (based on curve of machine learning word group search in PubMed). Data bases: Embase 1974 to 2022 August 16, MEDLINE(R) and Epub Ahead of Print, In-Process, In-Data-Review & Other Non-Indexed Citations and Daily 1946 to August 17, 2022.

Supplementary Table 2: Search strategy for Web of Science.

| **#** | **Searches** | **Results** |
| --- | --- | --- |
| #1 | TI=(hypoxia) OR AB=(hypoxia) OR TI=(hypoxic) OR AB=(hypoxic) OR TI=(hypoxemia) OR AB=(hypoxemia) OR TI=(hypoxemic) OR AB=(hypoxemic) OR TI=(hypoxaemia) OR AB=(hypoxaemia) OR TI=(hypoxaemic) OR AB=(hypoxaemic) | 151,732 |
| #2 | TI=("artificial intelligence") OR AB=("artificial intelligence") OR TI=("deep learn*") OR AB=("deep learn*") OR TI=("artificial neural network") OR AB=("artificial neural network") OR TI=("machine learning") OR AB=("machine learning") OR TI=("shallow learn*") OR AB=("shallow learn*") OR TI=("nearest neighbor") OR AB=("nearest neighbor") OR TI=("decision tree*") OR AB=("decision tree*") OR TI=("gradient-boosted tree*") OR AB=("gradient-boosted tree*") OR TI=("random forest*") OR AB=("random forest*") OR TI=("support vector machine*") OR AB=("support vector machine*") OR TI=("reinforcement learn*") OR AB=("reinforcement learn*") OR TI=("cluster analys*") OR AB=("cluster analys*") OR TI=("hierarchical cluster*") OR AB=("hierarchical cluster*") OR TI=("unsupervised learn*") OR AB=("unsupervised learn*") OR TI=("supervised learn*") OR AB=("supervised learn*") | 696,758 |
| #3 | #1 AND #2 | 475 |
| #4 | #3 NOT (TI=(wearable*) OR AB=(wearable*)) and English or German (Languages) and Marine Freshwater Biology or Oceanography or Plant Sciences or Fisheries or Zoology or Veterinary Sciences (Exclude – Web of Science Categories) | 435 |

Search date 09/08/2022. Time range: 2000 - current (based on curve of machine learning word group search in PubMed). Data bases: Web of Science Core Collection. No Medical Subject Headings (MeSH) supported.

Supplementary Table 3: Search strategy for Google Scholar using Publish or Perish.

| **#** | **Searches** | **Results** |
| --- | --- | --- |
| #1 | **Title Words:** "hypoxia" OR "hypoxic" OR "hypoxemia" OR "hypoxemic" OR "hypoxaemia" OR "hypoxaemic",  **Keywords:** "artificial intelligence" OR "deep learning" OR “artificial neural network" OR "machine learning" NOT "wearable" | 703 |
| #2 | **Title Words:** "hypoxia" OR "hypoxic" OR "hypoxemia" OR "hypoxemic" OR "hypoxaemia" OR "hypoxaemic",  **Keywords:** "shallow learning" OR "nearest neighbor" OR "decision tree" OR "gradient-boosted tree" NOT "wearable" | 184 |
| #3 | **in Title Words:** "hypoxia" OR "hypoxic" OR "hypoxemia" OR "hypoxemic" OR "hypoxaemia" OR "hypoxaemic",  **Keywords:** "random forest" OR "support vector machine" OR "reinforcement learning" OR "cluster analysis" NOT "wearable" | 807 |
| #4 | **in Title Words:** "hypoxia" OR "hypoxic" OR "hypoxemia" OR "hypoxemic" OR "hypoxaemia" OR "hypoxaemic",  **Keywords:** "hierarchical clustering" OR "unsupervised learning" OR "supervised learning" NOT "wearable" | 827 |
|  | Sum of #1 #2 #3 #4 without duplicates | 2090 |

Search date 09/07/2022. Time range: 2000 - current (based on curve of machine learning word group search in PubMed).

Supplementary Table 4: Data source of each study, for model development and external validation.

| **Study (year)** | **Data source** | | |
| --- | --- | --- | --- |
|  | **Model development** | **External validation** | |
| **Annapragada et al (2021)** | eICU data set | | JH-CROWN data set (Covid-19 patients) |
| **Chen et al (2021)** | AIMS of 2 US medical centers  MIMIC-III data set | | Additional medical center |
| **ElMoaqet et al (2014)** | Data from a US study assessing the reliability and nurse response times to a postoperative paging alert system (Voepel-Lewis et al 2013) | |  |
| **Erion et al (2017)** | AIMS data of an US academic medical center | |  |
| **Geng et al (2018)** | First Affiliated Hospital of Wenzhou Medical University, China | |  |
| **Geng et al (2019)** | First Affiliated Hospital of Wenzhou Medical University, China | |  |
| **Lam et al (2022)** | EHR data from 7 US hospitals | | Two different target hospitals |
| **Lundberg et al (2018)** | AIMS data of 2 US hospitals | |  |
| **Ren et al (2022)** | MIMIC-III data set | | University of Pittsburgh Medical Center (UPMC) cohort of 133 patients, US |
| **Sippl et al (2017)** | Clinical data warehouse of Erlangen University Hospital, Germany | |  |
| **Statsenko et al (2022)** | Al Ain Hospital, United Arab Emirates | |  |
| **Xia et al (2022)** | MIMIC-IV data set | |  |

Supplementary Table 5: Clinical variables used by each study.

| **Study (year)** | **Clinical variables** |
| --- | --- |
| **Annapragada et al (2021)** | Prior SpO2 values (two previous time points, 10 minutes prior data for SWIFT-5, 60 minutes prior data for SWIFT-30) |
| **Chen et al (2021)** | SAO2, ETCO2 end-tidal carbon dioxide, NIBP[S/M/D] non-invasive blood pressure (systolic, mean, diastolic), FIO2, ETSEV/ETSEVO end-tidal sevoflurane, ECGRATE heart rate from ECG, PEAK ventilator pressure, PEEP, PIP peak, inspiratory pressure, RESPRATE respiration rate, body temperature, height, weight, ASA code, ASA code emergency, gender, age |
| **ElMoaqet et al (2014)** | Prior SpO2 data (sampled every two seconds) |
| **Erion et al (2017)** | Prior SpO2 values (60 measurements, one per minute for the last hour of surgery) |
| **Geng et al (2018)** | Age, BMI, habitual snoring |
| **Geng et al (2019)** | BMI, habitual snoring, neck circumference |
| **Lam et al (2022)** | Age, sex, systolic blood pressure, diastolic blood pressure, heart rate, arterial partial pressure of oxygen, respiratory rate, peripheral oxygen saturation (required feature), temperature, glucose, bilirubin, white blood cell count, red blood cell count, lymphocytes, alanine transaminase, international normalized ratio, ph, blood urea nitrogen, creatinine, platelet, neutrophils, monocytes, hematocrit, lactate, aspartate aminotransferase, systemic inflammatory response syndrome score |
| **Lundberg et al (2018)** | 3905 real-time and static extracted features for each time point during anaesthesia care from the more than 20 original static sources and 45 different real-time data sources  Feature sources:  **Patient:** age, sex, ASA physical status ASA physical status of patient before surgery, height, weight, patient class (inpatient or outpatient)  **Procedure:** procedure description, surgical procedure code, billing codes (anaesthesia crosswalk/ procedure codes), diagnosis description, icd‐9/10 codes, operating room location, hospital facility, emergency status (whether case is an emergency or not – y/n), type of anaesthesia case events: time of anaesthesia start, time of patient in room, time of induction start, time of induction end or anaesthesia ready, time of procedure start (incision), time of closing, time of procedure end, time of start of emergence, time of leave or/ transport to recovery, time of anaesthesia end **Patient monitor/ventilator data:** heart rate from ECG signal, O2 saturation from pulse oximetry, pulse rate from pulse oximetry, NIBP – sys cuff BP – systolic, NIBP – dia cuff BP – diastolic, NIBP ‐ mean cuff BP – mean, arterial bp – systolic, arterial BP – diastolic, mean arterial BP – mean, pulmonary artery pressure – systolic, pulmonary artery pressure – diastolic, central venous line pressure – mean, end tidal CO2 (capnography), measured respiration rate – capnography, FiO2 inspired O2, end tidal sevoflurane anaesthetic agent, end tidal desflurane anaesthetic agent, end tidal isoflurane anaesthetic agent, et n20 end tidal nitrous oxide, bispectral index, signal quality index of bis, temperature, svo2 mixed venous oxygenation, CCO continuous cardiac output, tidal volume, ventilator rate setting, pip peak inspiratory pressure, peep positive end expiration pressure, O2 flow rate, air flow rate, N2O flow rate, type of cardiac rhythm  **Medications:** delivery time (start / end for infusion medications), drug name, drug dose, drug dose unit, drug route, fluid totals, time of fluid input or output, fluid name, fluid volume recorded, intraoperative laboratory results, time of taking sample or lab result time, lab result description, sample type – arterial/venous, result value, unit of measurement  **Notes:** time associated with the note, context code associated with a note or its selections, note description field |
| **Ren et al (2022)** | SpO2, FiO2, PEEP |
| **Sippl et al (2017)** | 17 different features in total  For multi-layer NN:  7 features: Area of the SaO2 curve below a presumed threshold of 95%-saturation, global drop of SaO2, latency between intubation and the observed SaO2 minimum, patient age, Cormack-Lehane scores of expected intubation difficulty, ‘awake intubation’ flag, and ratio of HR at time of SaO2 minimum to the global HR maximum  For random forest:  6 features: Basic patient attributes such as weight, but were mostly derived from the available time series, including mean arterial pressure, HR and SaO2) |
| **Statsenko et al (2022)** | Lung CT and 2D diagnostic images of the chest |
| **Xia et al (2022)** | RBC (max), PaO2 (final), WBC (min), pH (final), respiratory rate (final), lactate (max), PaO2 (min), heart rate (final), lactate (final), ventilation time, rbc (min), heart rate (min), PaCO2 (final), PaO2 (max), SpO2 (final), blood glucose (final), respiratory rate (max), SOFA (24 h), blood glucose (max), respiratory failure, SpO2 (min), mean airway pressure (min), SOFA CNS (24 h), PEEP (final), pneumonia, PSV level (final), gender, heart failure, vasopressor data |
